# Dynamics of Cytokine Profile Indicators Changes in Animals with Acute Generalized Peritonitis on the Background of Diabetes Mellitus

**DOI:** 10.25122/jml-2020-0005

**Published:** 2020

**Authors:** Ihor Yakovych Dzubanovsky, Svitlana Romanivna Pidruchna, Natalia Anatoliivna Melnyk, Serhiy Mykhailovych Andreychyn, Bogdana Mykhailivna Vervega, Natalia Anatoliina Nychyk

**Affiliations:** 1. Department of Surgery of Postgraduate Faculty, I. Horbachevsky Ternopil National Medical University, Теrnopil, Ukraine; 2. Department of Medical Biochemistry, I. Horbachevsky Ternopil National Medical University, Теrnopil, Ukraine; 3. Department of General Hygiene and Ecology, I. Horbachevsky Ternopil National Medical University, Теrnopil, Ukraine; 4. Department of Internal Medicine Propaedeutics and Phthisiology, I. Horbachevsky Ternopil National Medical University, Теrnopil, Ukraine; 5. Department of Pathological Physiology, I Danylo Halytsky Lviv National Medical University, Lviv, Ukraine; 6. Department of Infectious Diseases with Epidemiology, I. Horbachevsky Ternopil National Medical University, Теrnopil, Ukraine

**Keywords:** Cytokines, interleukins, acute generalized peritonitis, diabetes mellitus

## Abstract

In acute peritonitis, any surgical intervention leads to impaired immune protection with the development of postoperative purulent-septic complications, which increases several times the likelihood of death, especially in people with secondary immunodeficiency as a consequence diabetes mellitus.

We aimed to study the dynamics of pro- and anti-inflammatory cytokine content in rat serum under experimental acute generalized peritonitis on the background of diabetes mellitus.

Fifty-six white rats were used for the study. The determination of the serum cytokine profile was performed by enzyme-linked immunosorbent assay.

When comparing the levels of interleukins between the study groups, a statistically significant increase in the level of proinflammatory cytokines was found in the group of diabetic animals during all experimental periods. In particular, the concentration of interleukin – 1β increased significantly by 94% on day 1 of observation, by 115% on day 3, and by 121% on day 7 compared to the control group. Similarly, a significant increase in TNF-α levels was observed in animals with diabetes. In this group, the most significant increase in the level of TNF-α was recorded on the seventh day of the experiment, and it increased by 3.4 times.

Animals with acute peritonitis on the background of diabetes had a significantly increased concentration of anti-inflammatory cytokines in the serum of all study groups, which confirms their involvement in the pathogenesis of the disease under study.

## Introduction

Acute generalized peritonitis (AGP) is increasingly associated with concomitant diabetes mellitus (DM), resulting in changes in its mechanisms of development and worsening of the effects of treatment. However, any surgery leads to impaired immune protection with the development of postoperative purulent-septic complications [1-2], which increases several times the likelihood of death, especially in people who have secondary immunodeficiency on the background of chronic diseases, metabolism disorders, and endotoxicosis. In the early postoperative period, there are already pronounced disorders of phagocytosis, cellular and humoral immunity.

Currently, peritonitis is considered in the inflammatory process dynamics, which is characterized by the release of pro- and anti-inflammatory cytokines [3-4]. The main proinflammatory cytokines are tumor necrosis factor-alpha (TNF-α), interleukin (IL)-1 β, IL-6, and IL-8. TNF-α and IL-1 β are early regulators of the immune response and induce the release of secondary cytokines such as IL-6 and IL-8 [5-6]. As peritonitis progresses, cytokine production occurs in accordance with the stages of the pathological process: from activation of synthesis with a massive cascade release of cytokines to inhibition of this process with the development of immunoparalysis. Further progression of purulent inflammation in the abdominal cavity with the addition of other sources of endogenous intoxication (including the development of enteric insufficiency) leads to hyperactivation of macrophages, neutrophils, endothelial system, T- and B lymphocytes [7-8]. In this regard, the content of cytokines in the blood and the cells that produce them sharply increases. Then, an imbalance develops between inflammatory mediators and mechanisms that control their production toward the overproduction of cytokines, eicosanoids, reactive oxygen species (NO-, O2-, OH-, H2O2, ONO2-), stress hormones, and amino peptides. Cytokines circulating in the blood continuously activate new cells with the emergence of a cascade (uncontrolled) reaction with their hyperproduction (the so-called “cytokine storm”). Thus, there are systemic manifestations (syndromes) of toxic action of cytokines – the syndrome of “leakage of capillaries”, syndrome of septic shock, and others. They are accompanied by impaired microcirculation, pronounced vasodilation, venous overflow, increased vascular wall permeability, and edema, hypovolemia, tissue hypoxia, decreased blood pressure, fever, or metabolic acidosis [9-10]. Under certain conditions, these changes translate into sepsis, septic shock, and irreversible multiple organ failure.

Thus, cytokines can play a significant role in the pathogenesis of AGP on the background of DM, but the specific mechanism of their action is not yet well understood. Therefore, the aim of our work was to study the content of pro- and anti-inflammatory cytokines in the serum of rats under the conditions of experimental AGP on the background of DM.

## Material and Methods

The experiment involved 56 white rats, which were divided into three groups: the main group – 24 animals with simulated AGP on the background of DM; comparison group – 24 animals with simulated AGP; the control group – 8 intact animals, which were kept under standard vivarium conditions. All groups of animals compared were representative in terms of weight, sex, and age.

The experimental DM was reproduced by intraperitoneal administration of streptozotocin on an empty stomach at a dose of 60 mg/kg (“Sigmal”), which was dissolved in sodium citrate buffer, pH 4.5) [11]. Studies on the glucose content were carried out by the glucose-oxidant method at 9:00h under conditions of free access of experimental animals to food and water during the night period. Insulin (0-2 units subcutaneously, two to five times a week) was administered to rats throughout the observation period.

After two weeks from the use of streptozotocin in rats, the glucose content from the venous blood, which was obtained from the tail vein, was determined. In subsequent studies, only those rats in which the glucose content was greater than 300 mg/L were further examined. Animals of the control group were administered subcutaneously sterile sodium chloride solution 0.9%.

The influence of AGP on the course of DM was studied on the model proposed by Lazarenko et al. [12]. This model is close to a similar process in humans in terms of etiological factors, clinical manifestations, and course phase. On the 14th day after the administration of streptozotocin, animals of the main group were injected with 10% filtered fecal suspension into the abdominal cavity at a dose of 0.5 ml per 100 g of body weight. Rats of the comparative group received only a subcutaneous injection of fecal suspension. The fecal suspension was obtained by mixing an isotonic solution and feces from the cecum of 2–3 intact animals, then filtered twice through a double layer of gauze. The resulting suspension was injected into the intact rats in a puncture manner no later than 20 minutes after preparation. In order to avoid damage to the internal organs when the fecal suspension was introduced into the abdominal cavity, the animals were kept upright. By the method of puncture of the ventral wall in the center of the midline of the abdomen, directing the end of the needle alternately into the right and left hypochondrium, right and left iliac areas, the same amount of fecal suspension was administered.

The observation dates were 1, 3, and 7 days. This experimental study was conducted in accordance with the general rules and regulations of the European Convention on the Protection of Vertebrate Animals, which are used for research and other scientific purposes (Strasbourg, 1986).

Determination of cytokine profile in serum (interleukin-1β (IL-1β), interleukin-10 (IL-10) and tumor necrosis factor-α (TNF-α)) was determined by enzyme-linked immunosorbent assay using an “ELISA Kit for Rats”, Uscn Life Science Inc. according to the manufacturer’s instructions [13].

## Results

Currently, there is no doubt that in AGP against the background of DM, the activation of specific and nonspecific immune responses is associated with the effect on various homeostatic systems of a variety of universal mediators, among which cytokines are essential. They are often called cellular hormones because they interact with specific cellular receptors, exerting autocrine, paracrine, and endocrine regulation. Cytokines act primarily not only at the level of the inflammation site and the territory of the responding lymphoid organs, but also at the level of the whole organism, so there is a need to study the interleukins in the blood plasma to study the systemic nature of inflammation in AGP against the background of DM.

**Figure 1:**
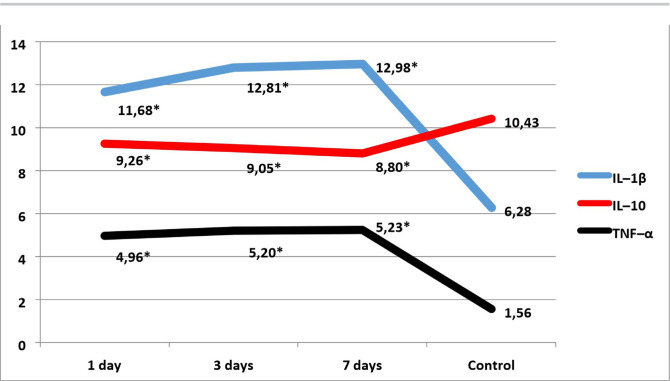
Levels of pro- and anti-inflammatory serum interleukins in animals with AGP. Notes: * – significance of the difference of indicators in comparison control group.

**Figure 2:**
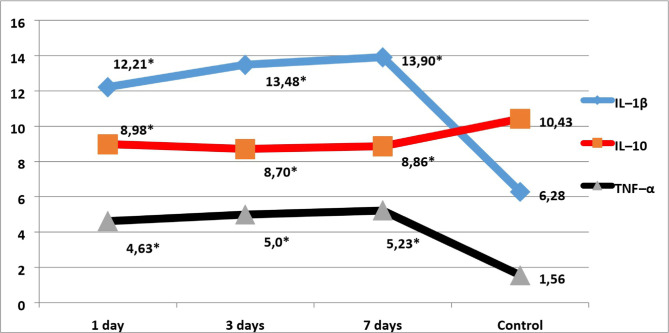
Levels of pro- and anti-inflammatory serum interleukins in animals with AGP against the background of DM . Notes: * – significance of the difference of indicators in comparison to the control group.

Our study showed that on the first day after the modeling of AGP (comparative group), the level of IL-1β in serum increased statistically significantly by 86%. In contrast, on the third day, there was a significant increase of 104% relative to intact animals and 107% compared to the control group on day 7 of the experiment (Figure 1). In animals of the main experimental group with AGP against the background of DM, the concentration of IL-1β significantly increased on 1 day of observation by 94%, on the third day by 115%, on the seventh day by 121% compared with the intact group (Figure 2).

In the analysis of the anti-inflammatory IL-10 level on days 1, 3 and 7, it was noted that in the serum of experimental animals with AGP, there was a statistically insignificant decrease of this cytokine by 11%, 13% and 16%, respectively. The study showed that the level of IL-10 in the serum of animals with AGP on the background of DM significantly decreased by 14% in the first day, by 17% on the third day, by 15% on the seventh day compared to intact animals (Figure 2). 

In our study, the level of TNF-α in the serum of a group of animals with simulated AGP for the first, third and seventh days of the experiment increased statistically significantly by 3.2, 3.3 and 3.4 times, respectively. Similarly, the indicator of growth was observed in the animals of the main group. The most significant increase in TNF-α level in animals of the AGP group against the background of DM was recorded on the seventh day of the experiment – it increased by 3.4 times.

Analyzing these results, we can conclude that modeling of AGP against the background of DM leads to impaired immunological and nonspecific reactivity of the organism.

## Discussion

Analyzing these results, we can conclude that modeling of AGP against the background of DM leads to impaired immunological and nonspecific reactivity of the organism. It is known that the microbial factor in peritonitis is the trigger mechanism of subsequent pathophysiological disorders [14]. Under the influence of microorganisms and their toxins, the cells of the immune and reticuloendothelial systems (tissue macrophages, endothelial cells, polymorphonuclear neutrophils, basophils, mast cells, and lymphocytes) are activated, and a local inflammatory reaction first develops. During this period, local production of activated cells by various cytokine mediators is noted: interleukins, tumor necrosis factors, interferons, growth factors [15].

The initiation and major stages of the development of an inflammatory response are controlled mainly by proinflammatory cytokines produced by macrophages, neutrophils, and T-cells in response to stimulation by bacterial agents. Naturally, gram-negative endotoxin stimulates macrophages and neutrophils to produce TNF- α, IL-1, IL-12, IL-8, and IL-6. Exotoxins, superantigens of gram-positive bacteria activate T-cells and monocytes to produce IL-2, IL-1, TNF-α. At the same time, proinflammatory cytokines play a protective role because they provide infections with effector cells (neutrophils, macrophages), stimulate their phagocytic, bactericidal activity, and induce the initiation of an antigen-specific immune response, which together promotes pathogen elimination [16].

Due to the massive contamination of the abdominal cavity and the formation of a large number of toxins and biologically active substances that interact with the highly reactive and highly resorptive peritoneum, the mechanisms of limiting the local inflammatory process in conditions of peritonitis are widespread. As a result of increasing the permeability of the endothelium, cytokines enter the systemic bloodstream, where they activate leukocytes and platelets and cause the release of adhesion molecules from the endothelium. Initially, under the action of a small number of proinflammatory cytokines (IL-1, IL-6), an initial acute-phase reaction occurs, aimed at mobilizing the body’s defenses at the systemic level [17]. This process is part of the general adaptive response of the body to inflammation and is characterized by activation of the neuroendocrine system, stimulation of leukocytopoiesis in the bone marrow and the release of neutrophils into circulation from the bone marrow, increased hepatic production of acute-phase proteins, development and generalized. At this stage, the compensatory secretion of anti-inflammatory mediators (IL-4, IL-10, IL-13) counteracts the overactive activity of proinflammatory cytokines. Further progression of peritonitis is accompanied by increased absorption of purulent effusion derivatives from the abdominal cavity, and progressive bowel paresis and ischemia of the intestinal wall causes translocation of the intestinal flora and the intake of microorganisms and their toxins into the intestinal bloodstream. This leads to over-activation of cytokine-producing cells, massive production and accumulation of cytokines in the blood. In such conditions, proinflammatory cytokines, together with other inflammatory mediators, become aggression factors, exhibiting a destructive effect on the tissues and endothelium, and as a consequence, inflammatory reaction syndrome and abdominal sepsis develop [18]. TNF, a key multifunctional cytokine of systemic action, plays a dominant role in the development of local and general pathological processes. In high concentrations, it delivers a powerful cytotoxic effect, induces cell necrosis, impairs the permeability and function of the endothelium of the capillaries, which leads to a disorder of microcirculation and hypoxia of tissues, increases oxidative stress and catabolism processes, causes central hypertension, synthesis of other cytokines, as confirmed by our research.

It is also known that DM is characterized by a progressive deterioration of pancreatic function [19], resulting in intensified beta-cell apoptosis. It is known that IL-1β may mediate beta-cell apoptosis. IL-1β binds to the IL-1 receptor on the surface of these cells, leading to the activation of pro-apoptotic NF-κB transcription factors, DNA fragmentation, and loss of functional activity. IL-1β activates ІкВ βkinases and thus may induce insulin resistance.

Thus, interleukin-1 β may be one of the factors that differentially affect the nature of pancreatic β-cell function - from stimulation to inactivation, which is characteristic of the dynamics of DM development. At the same time, the metabolic shifts that accompany this disease, in particular hyperglycemia [20], are the cause of long-term persistence of increased activity of cytokine-producing cells and thus contribute to the progression of the disease. It was found that under the conditions of experimental AGP against the background of DM, the level of IL-1 in the serum on day 1 increased by 94% compared with the control animals.

The data obtained indicate the activation of macrophages and the possibility of inhibition of β-cell functional capacity. IL-1β activates T and B lymphocytes, enhances their cytotoxic properties, and initiates TNF-α synthesis.

As a typical anti-inflammatory cytokine, which plays a key role in the regulation of the intensity of inflammation and the effectiveness of immune defense, TNF-α refers to markers of nonspecific generalized inflammation. It has numerous immunomodulatory effects that are fundamental in the pathogenesis of autoimmune inflammation. Currently, there is evidence that TNF-α has the ability to stimulate the proliferation of autoreactive lymphocytes of different classes, to cause aberrant expression of adhesion molecules and antigens of histocompatibility class II on the surface of pancreatic cells, which can lead to the disruption of the process of antigen recognition [21]. In addition, one of the key biological properties of TNF-α is its involvement in the receptor pathway of apoptosis of immunocompetent cells.

Changes in the TNF-α system in diabetes can be explained from two points. First, they can be a consequence of metabolic disorders (dysglycemia, dyslipidemia [22] in diabetes [23], and, as a consequence, “metabolic immunodeficiency”, in which it is necessary to increase the integral indicators of the functional properties of mononuclear leukocytes due to T-cell production to maintain a normal immune response [24]. The second hypothesis is that abnormalities in the TNF-α system are primary, genetically determined, and, along with other mechanisms, cause the immunopathogenesis of autoimmune diabetes.

IL-10 is a typical anti-inflammatory cytokine that is able to block the synthesis of cytokines produced by macrophages, such as proinflammatory cytokines (interferon and TNF-α) [25]. Therefore, it is advisable to believe that IL-10 has a protective effect on diabetes. We recorded a decrease in the content of the investigated cytokine in animals of all study groups, at all times, which is quite natural and consistent with literature data.

## Conclusion

In animals with AGP on the background of DM, the concentration of anti-inflammatory cytokines in the serum of animals of all study groups was significantly increased, which confirms their involvement in the pathogenesis of the investigated pathology. In particular, the concentration of interleukin – 1β increased significantly by 94% on the first day of observation, by 115% on the third day, and by 121 on the seventh day compared to the control group. Similarly, a significant increase in TNF-α levels was observed in animals with diabetes. The most significant increase in the level of this indicator in this group of animals was recorded on the seventh day of the experiment – by 3.4 times.

Under these conditions, a new level of regulatory interconnection is emerging, where changes in the cytokine system of macrophage origin play an important role. This suggests that the development of complications in this comorbid pathology is associated with a violation of nonspecific immunity.

## Acknowledgments

The work is a fragment of the planned research work – Development of new open (miniaccess) and laparoscopic surgical interventions in the treatment of diseases of the abdominal cavity on the principles of the multimodal program “fast track surgery” (State registration number 0119U002805). 

## Conflict of Interest

The authors declare that there is no conflict of interest.
